# Factors influencing the selection criteria of Saudi board orthopedic surgery applicants: a national survey of program directors

**DOI:** 10.1186/s12909-023-05007-4

**Published:** 2024-01-12

**Authors:** Suhail S. AlAssiri, Alwaleed A. Alshahir, Sami I. Al Eissa, Fahad H. Al Helal, Faisal M Konbaz, Majed S. Abaalkhail, Rayed M. Al Jehani, Abdullah F. Mohabbat, Khalid A. AlSheikh

**Affiliations:** 1 Orthopedics Surgery Department, Ministry of the National Guard-Health affairs, King Abdulaziz Medical City, PO Box 22490, 11426 Riyadh, Saudi Arabia; 2 King Abdullah International Medical Research Centre, Riyadh, Saudi Arabia; 3 King Saud bin Abdulaziz University for Health Sciences, Riyadh, Saudi Arabia; 4 King Faisal Specialist Hospital and Research Centre, Riyadh, Saudi Arabia; 5 Spine Surgery Department, Neuroscience Centre, King Salman Bin Abdulaziz Medical City, Ministry of Health, Al Madina, Saudi Arabia

**Keywords:** Education, Orthopedics, Program directors, Medical students, Saudi Arabia, Residency selection

## Abstract

**Background:**

Annually, medical students vie to secure a seat with an orthopedic residency program. This rigorous competition places orthopedic surgery as one of the most competitive specialties in the medical field. Although several international studies have been published regarding the factors that influence program directors when choosing their ideal applicant, the data for Saudi Arabia in that regard is absent.

**Methods:**

In this cross-sectional study, we aimed to survey all orthopedic program directors regarding the factors that influence them when choosing their ideal orthopedic surgery applicant. A survey was sent to all program directors via email during the month of August 2022. A reminder was sent 2 weeks later to maximize the response rate. The survey was completed by 22 out of 36 orthopedic program directors, which gave us a response rate of 61.11%.

**Results:**

In this study, 22 orthopedic surgery program directors responded to our survey. When program directors were asked to rank the factors of residency selection criteria, the top ranked factors were good impression on interviews; prior experience in orthopedic surgery with, for example, electives; and performance on ethical questions during interviews, with means of 9.18, 8.95, and 8.82 out of 10, respectively. Furthermore, program directors preferred letters of recommendation from recommenders that they personally know, clinical experience such as electives taken at the program director’s institution, and the quality of publications as the most important aspects of research. Most program directors (90.9%) relied on their residents’ and fellows’ opinions when selecting candidates, and 77.3% did not think gender has an influence on selection of applicants.

**Conclusion:**

By providing comprehensive data regarding the factors that influence and attract program directors of orthopedic surgery when choosing residency candidates. With the data provided by this study, applicants for orthopedic surgery have the advantage of early planning to build a strong application that may help persuade program directors to choose them.

## Introduction

Annually, medical students vie to secure a seat inan orthopedic residency program. This rigorous competition places orthopedic surgery as one of the most competitive specialties in the medical field [1, 2]. In the match for 2021, only 866 applicants out of 1,289 in the United States were matched to orthopedic surgery programs [2]. This level of competition is not limited to the United States alone. According to data provided by the Saudi Commission for Health Specialties (SCFHS), in 2021, only 115 of the 175 applicants to orthopedic residency in Saudi Arabia were matched.

Considering this fierce competition, previous studies conducted globally have explored the criteria by which program directors select their applicants to offer a simplified approach for medical students in understanding what is important for residents’ selection [3, 4]. However, certain areas have not been extensively studied, such as residents’ and fellows’ feedback [5–7]. Hence, the absence of information regarding this topic locally, along with increasing competition, creates an overwhelming environment for medical students. This puts the students under tremendous pressure to meet the requirements necessary to ensure a spot in their preferred specialty while having uncertainty about which factors will increase their chances of acceptance.

The Saudi match system starts when applicants rank their desired specialty in the SCFHS completely computerized central matching system. Applicants are allocated to their desired specialty in their preferred region of the country for interviews based on a formula that takes into account their cumulative score on the Saudi medical license exam (SMLE) (55%), grade point average (GPA) during medical school (30%), and on elements that are assigned as points on the curriculum vitae (15%) [8].

To the best of our knowledge, no previous studies have explored the factors by which orthopedics surgery program directors choose their applicants in Saudi Arabia. Hence, our aim was to identify the characteristics that orthopedic surgery program directors in Saudi Arabia look for when choosing an ideal applicant.

## Materials and methods

In this cross-sectional study, we aimed to survey all Saudi Arabian orthopedic program directors on the factors that influence them when choosing their ideal orthopedic surgery applicant. A survey was sent to all program directors via email during the month of August 2022. A reminder was sent 2 weeks later to maximize the response rate. The survey was completed by 22 out of 36 orthopedic program directors, giving a response rate of 61.11%.

### Eligibility criteria

All orthopedic program directors were included in this study, while program directors from other specialties were excluded.

### Questionnaire

The items relating to our questionnaire were collected from primarily two studies in the literature, with several additional questions included by the authors [3, 9]. After formulating the questionnaire, we sent it to five experts in the field, including one program director, to obtain their feedback regarding the questionnaire’s clarity and relevance. Additionally, the survey was face and content validated after it was sent to an additional ten experts.

### Measures

The questionnaire first explored the demographic data of the respondents. Then, the respondents were asked to give a number of importance between 0 (not important) to 10 (utmost importance) on items relating to clinical, academic, and personal traits. These included GPA, SMLE score, good impression on interviews, clinical experience in orthopedic surgery, research experience, attainment of a post-graduate degree, presentation of research at conferences, inability to get into the specialty on first attempt, letters of recommendation (LOR), performance on ethical questions, and attendance at courses and conferences related to orthopedic surgery. The latter questions were in yes/no format and assessed the aspects of importance regarding the LOR, research, clinical experience in the field, passing international examinations, preference between recent and older graduates, gender preference, reputation of applicant’s medical school, winning awards or honors, having knowledge in orthopedic surgery, importance of residents’ and fellows’ opinions, and whether applicants would be ranked solely on their interview performance. The final part assessed preferences regarding the interview modality, limitations of implemented virtual interviews in several interview committees, and satisfaction with the current selection process in Saudi Arabia.

### Statistical analysis

Initially, the data were exported from a Microsoft Excel spreadsheet (Microsoft Corp., Redmond, WA, USA) to IBM SPSS Statistics for Windows, Version 26.0 (IBM Corp., Armonk, NY, USA). Demographic data, multiple choice questions, and yes/no questions were calculated using frequency, while the ranking of factors relating to residency selection criteria were given a mean score.

## Results

The current study included 22 orthopedic surgery program directors. The mean ± SD age of program directors was 41.1 ± 4.8. Among the 22 program directors, only 1 (4.5%) was female. Regarding their experience, 9 (40.9%) had spent less than 2 years as a program director. As for the distribution of program directors across Saudi Arabia’s regions, 11 (50%) served in the central region, 5 (22.7%) in the western region, 5 (22.7%) in the eastern region, and 1 program director was from the northern region. Program directors working in Ministry of Health hospitals constituted 40.9% of the sample, followed by military hospitals, university hospitals, and Security Force hospitals, each of which constituted 13.6%. More demographic information is provided in Table 1.

**Table 1:** Demographic data

**Parameter**	**Category**	**No. (%)**
Age	NA	41.1 ± 4.8
Gender	Male	21 (95.5%)
	Female	1 (4.5%)
Years of experience as program director	Less than 2 years	9 (40.9%)
	2–5 years	8 (36.4%)
	More than 5 years	5 (22.7%)
Region of service	Central	11 (50%)
	Western	5 (22.7%)
	Eastern	5 (22.7%)
	Northern	1 (4.5%)
Workplace	Ministry of Health	9 (40.9%)
	Military hospitals	3 (13.6%)
	University hospitals	3 (13.6%)
	King Faisal Specialist Hospital and Research Centre	2 (9.1%)
	National Guard Health Affairs hospitals	2 (9.1%)
	Security Forces hospitals	3 (13.6%)

When participants were asked to rank residency selection criteria, the factors of good impression on interviews; prior experience in orthopedic surgery, such as with electives; and performance on ethical questions during interviews were the top factors, with means of 9.18, 8.95, and 8.82 out of 10, respectively. The complete ranking of residency selection criteria is presented in Table 2.

**Table 2:** Results of program director rankings from the questionnaire

**Rank**	**Residency Selection Criteria**	Program Directors, Mean*
1	Good impression in an interview	9.18 ± 1.09
2	Prior experience in orthopedics, such as with electives	8.95 ± 1.39
3	Performance on ethical questions during interviews	8.82 ± 1.86
4	Experience and knowledge in research demonstrated by publications and courses	7.82 ± 0.90
5	Presenting posters or oral presentations at events	7.82 ± 1.86
6	Letters of recommendation	7.32 ± 2.12
7	Attending courses and conferences related to orthopedic surgery	7.32 ± 2.14
8	SMLE score†	6.91 ± 2.50
9	GPA†	6.86 ± 1.95
10	Attaining a post-graduate degree	5.55 ± 6.50
11	Inability to get into an orthopedic program from initial attempt	5.14 ± 2.80

^*^The values are given as the mean and the SD; † = with the assumption that the applicant passed the minimum required from the SCFHS

**Fig. 1:**
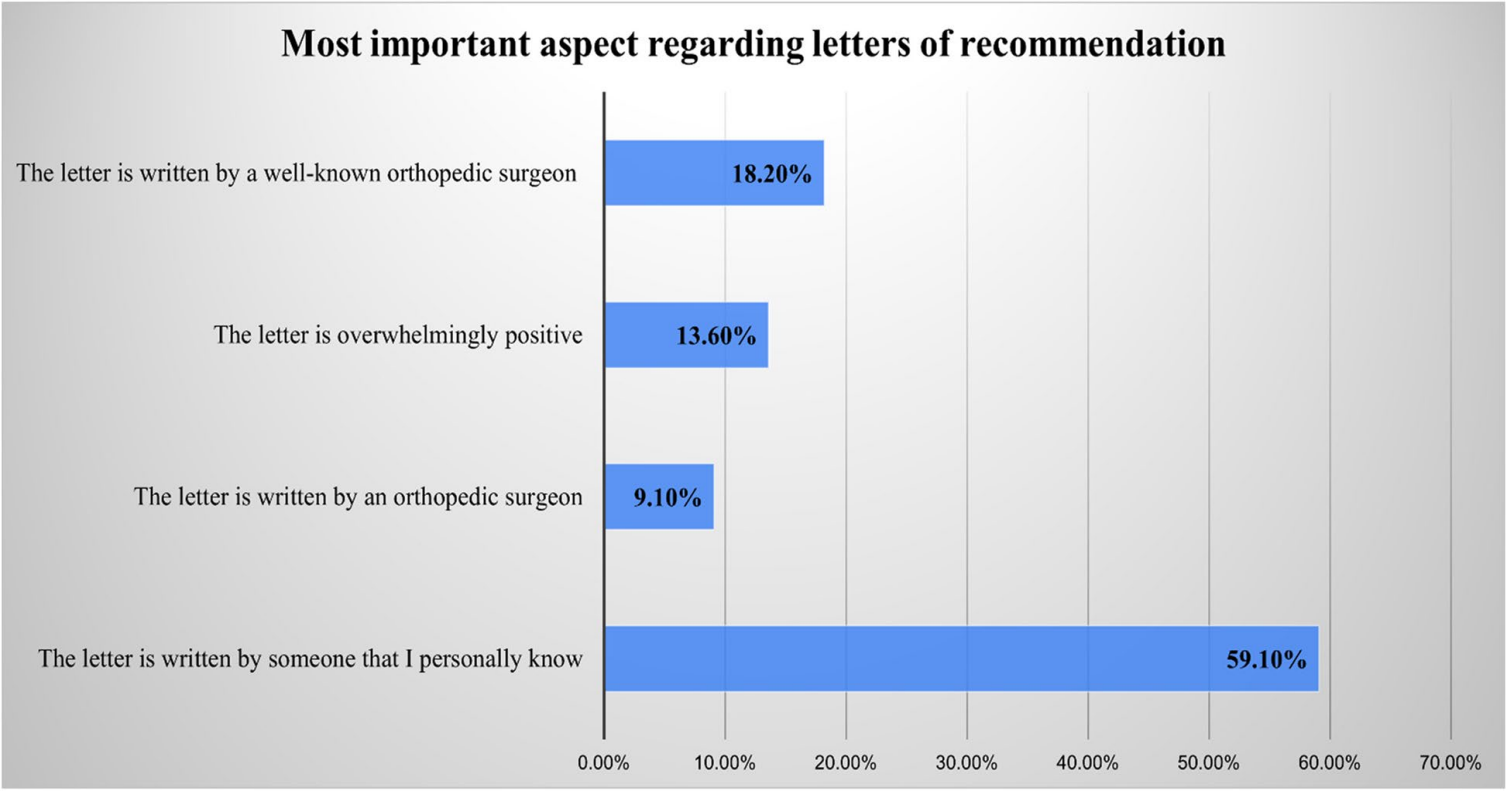
Responses to the question, “Which of the following aspects regarding recommendation letters is the most important?”

**Fig. 2:**
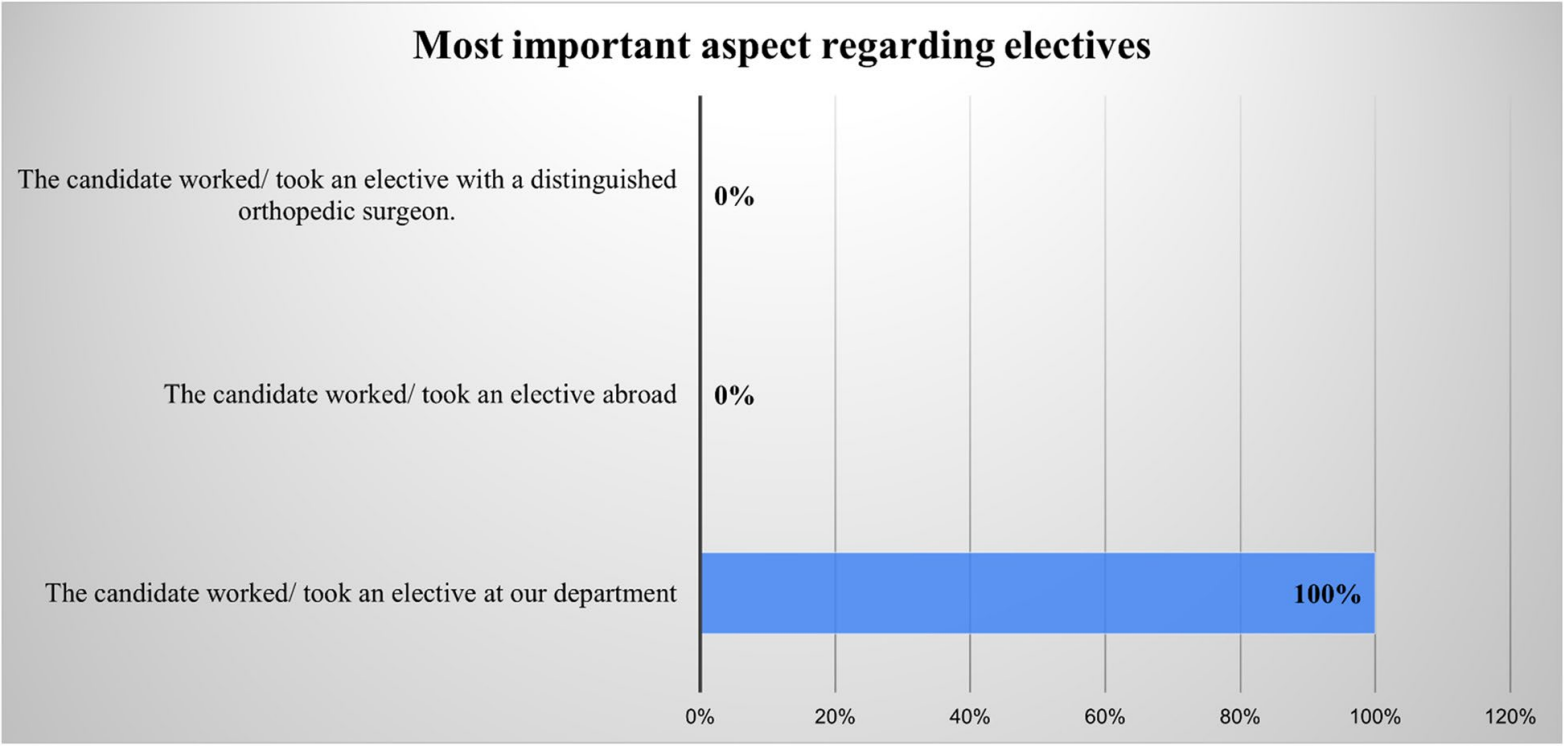
Responses to the question, “Which of the following aspects regarding orthopedic clinical experience is the most important?”

**Fig. 3:**
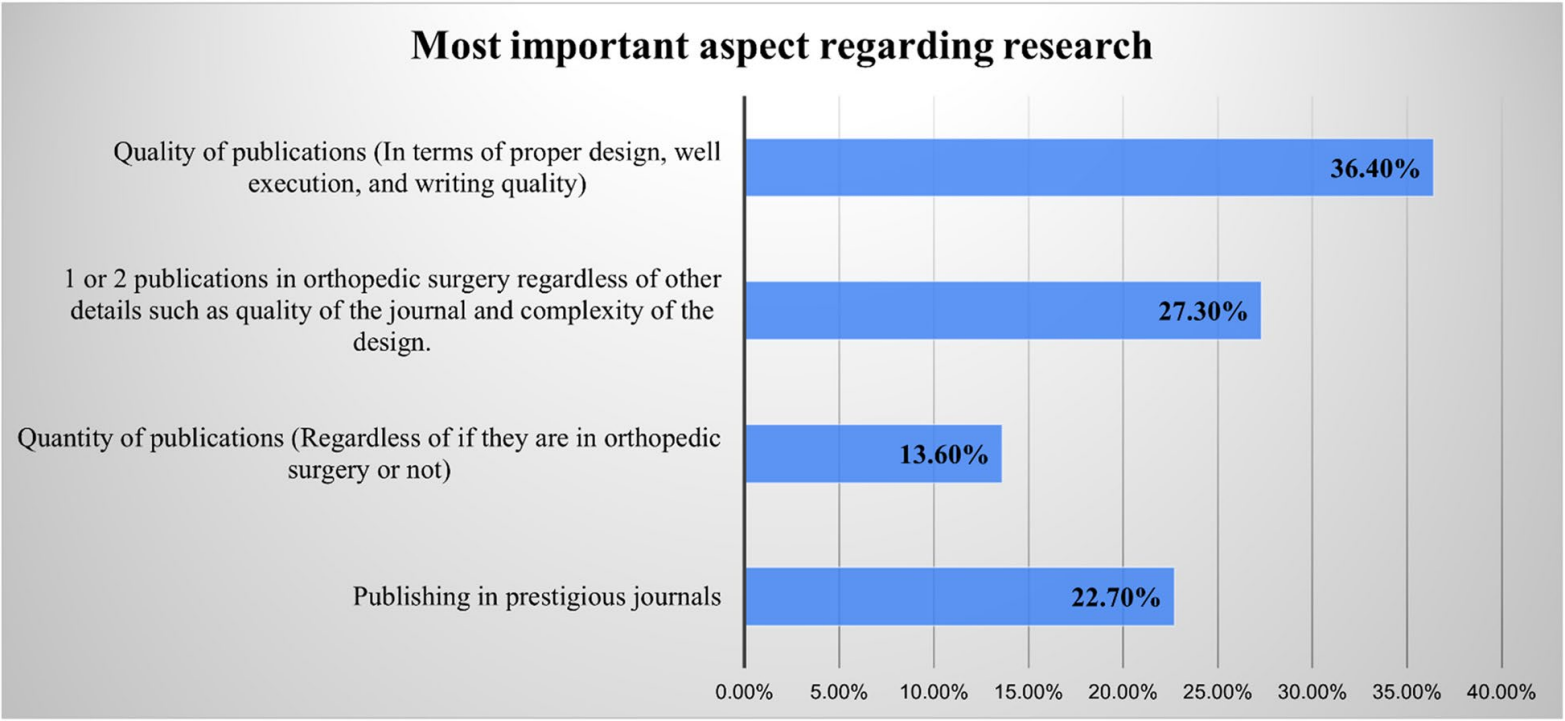
Responses to the question, “Which of the following aspects regarding research is the most important?”

The results of the multiple choice questions pertaining to the most important aspects of the LOR, orthopedic clinical experience, and research are displayed in Figs. 1, 2, and 3. In addition, the results of the yes/no questions showed that program directors value applicants with awards and honors (yes = 59.1%), prefer applicants who possess knowledge in orthopedic surgery (yes = 54.5%), and rely heavily on their residents’ and fellows’ opinions (yes = 90.9%). These responses also showed that most program directors have no increased preference for applicants who have passed international examinations, such as USMLE, or who attended a reputable medical school, and they have no preference regarding the applicant’s gender. Table 3 provides the program directors’ answers to all the yes/no questions.

**Table 3:** Results of the yes/no questions from the program directors survey

**Item**	**Program Director Responses (%)**
Is research experience in basic sciences more impressive than the clinical field?	Yes (50%)
Once selected for an interview, are all candidates considered equal for the final decision made based solely on the candidate’s performance during the interview?	Yes (54.5)
Is an applicant who passed international licensing examinations, such as USMLE, more likely to be accepted in your program?	No (95%)
Does an applicant who graduated within the last 2 years have a better chance of acceptance in your program?	Yes (50%)
Is the gender of the applicant important for acceptance in your program?	No (77.3%)
Is the reputation of the applicant’s medical school important for acceptance in your program	No (63.6%)
Are awards and honors important when selecting an applicant?	Yes (59.1%)
Does knowledge in orthopedic surgery play a significant role in accepting an applicant?	Yes (54.5%)
Do you rely on your residents’ and fellows’ opinions in selecting an applicant?	Yes (90.9%)

The last part of the survey examined interview preferences and overall satisfaction with the current matching process. Most of the program directors held interviews in person with applicants (59.1%). In addition, almost all program directors preferred interviews to be done in person (95.5%), and 81.8% believed that virtual interviews limit the selection of applicants. The response to overall satisfaction with the current matching process is presented in Table 4.

**Table 4:** Results of program directors’ answers on interview preference and selection process

**Question**	**Program Director Response**
**Question 1**	
How is the interview done at your program?	
In person	59.1%
Virtually	9.1%
Mixed	31.8%
**Question 2**	
Do you prefer interviews to be done in person or virtually?	
In person	95.5%
Virtually	4.5%
**Question 3**	
Do you believe that virtual interviews are limiting your resident selection?	
Yes	81.8%
No	18.2%
**Question 4**	
What is your satisfaction with the current selection process?	
Very satisfied	27.3%
Somewhat satisfied	31.8%
Neutral	18.2%
Somewhat dissatisfied	22.7%
Very dissatisfied	0.0%

## Discussion

The findings of this study show that program directors place great emphasis on applicants who performed well on interviews, had clinical experience at the director’s institution, and demonstrated good knowledge regarding ethical questions. Furthermore, program directors stated that they relied significantly on their residents’ and fellows’ opinions regarding applicants and desired an applicant with awards and honors. In contrast, factors such as the gender of the applicant, reputation of the applicant’s medical school, or passing international examinations such as USMLE were believed to be not important to program directors for selecting applicants.

In the Saudi match, all applicants must pass a two-step match in order to secure a seat in a residency position. The first match, which is computerized, is done by ranking applicants based on a cumulative score of 55% for the SMLE, 30% for GPA during medical school, and 15% on elements that are assigned as points on the curriculum vitae. The second match, which is considered the final judgment on whether an applicant is accepted, is when interviews take place [8]. An applicant’s performance on interviews was highly valued by program directors in previous studies [3–5, 10]. Our study showed similar results; out of the important factors discussed in this study, the applicant’s impression during the interview was the deciding factor, with a mean of 9.18 out of 10 for importance in the selection process. However, it must be noted that the interview is not an isolated event, and that having good LOR, performing well on electives, and having done research also play a role in persuading the committee to think highly of applicants before interviewing them.

Electives or away rotations were among the most important deciding factors for acceptance according to many program directors. Previous studies surveying program directors of orthopedic surgery have demonstrated that performance during a rotation at the program director’s institution was the most important factor in selecting applicants [3–6, 10]. Similarly, this study showed that program directors gave more weight to electives in accepting applicants, particularly when these electives were completed at the program director’s institution. In fact, all respondents to our survey preferred an applicant who had done a rotation at their institution. In the local literature, performance during rotations was the deciding factor in Alyami et al.’s survey of urology program directors and was the second most important factor in a survey of plastic surgery program directors [9, 11].

Although research is thought by medical students to be an important factor in resident selection, this notion was contrary to what was presented in the global literature. In their retrospective analysis of orthopedic surgery applicants’ publication statuses, Ngaage et al. showed that the actual average publication was 2.6 to 6.6 and a mean of 1 publication per applicant, which was lower than that reported by the national residency matching program (NRMP) [12]. Similarly, a cross-sectional survey of orthopedic program directors by Bram et al. showed that only 3 (4%) of the program directors considered that taking a year-out program in research would increase the applicant’s chances of acceptance [13]. Rivero et al. also studied the outcomes of a year-out program on residency selection, and the results showed similar outcomes as in Bram et al. that those who completed a research year-out program did not have greater chances of acceptance [13, 14]. In a study by Campbell et al., the researchers demonstrated that more publication is important in institutions that have a dedicated research center. These institutions valued applicants with prior research experience more [15]. Nevertheless, the emergence of research has not been long established locally. This notion can be looked at in light of Al-Mohrej et al.’s study, which discussed how the level of research participation and knowledge among orthopedic residents in Saudi Arabia was low [16]. Likewise, in a study comparing a Canadian orthopedic program with a Saudi program, the results showed lower numbers of research participation and article publication among the Saudi residents [17]. Therefore, the importance of research in the orthopedic field was exemplified by the Saudi orthopedic committee when a new promotion role mandated the resident to have a research project. Nowadays, research plays a major role in acceptance in Saudi board programs. This was demonstrated in our study results, where major emphasis was given to research knowledge, which the applicant must display by publications, courses, or presentations at conferences. In addition, research is also considered important because it is a project that can be extensive and applicants must work as part of a team and may face several obstacles while working in such projects, hence, direct supervision will unveil several traits that can be demonstrated by applicants such as teamwork, attitude, adherence to deadlines, and handling criticism. Thus, research can allow an extensive and thorough assessment of applicants by their supervisors.

This study showed that LORs are an effective tool if appropriately used by applicants. Program directors in this study valued an LOR from orthopedic surgeons they personally know. Similarly, studies by Bernstein et al. and McDonald et al. discussed the significance of an LOR by asking program directors about the important aspects of an LOR. The most important aspect to program directors was that the LOR is written by someone they know [3, 4].

Applicants to orthopedic surgery tend to work synergistically and closely with residents and fellows; thus, their opinions are an asset to be taken into consideration for a holistic view of the applicant. Despite the importance of this input and the ability to steer the committee of orthopedics in selecting residents, to our knowledge, only McDonald et al. has assessed this factor in their survey of program directors [4]. Their study indicated that 89% of program directors relied on residents’ input. Similarly, 90.9% of program directors in this study put great emphasis on their residents’ and fellow’s feedback. This shows that residents’ and fellows’ opinions are an integrated and important part of the selection process that applicants need to be aware of.

This cross-sectional study explored and reviewed the perspectives of orthopedic program directors in choosing their applicants. The strength of this study is that it provides comprehensive empirical data regarding what program directors value in choosing their applicants, which to our knowledge had not previously been present in the local literature.

This study also faced some limitations. First, the response rate was less than anticipated, with 22 program directors out of 36 completing the survey for a response rate of 61.11%. Another limitation is that this study’s data cannot be generalized to all interview committees because other faculty can add weight to decisions about applicant selection, such as department heads or deputy program directors. In addition, there is a possibility of conformity bias in this study, which is a natural part of any survey. Another limitation in this study is not addressing the negative factors that applicants may present with including but not limited to ethical concerns, poor attitude, and negatively worded LOR. Such factors when present can significantly impact the decision making of the selection committee.

Hence, Future studies should address the impact of negative factors on applicants’ selection as well as the essential skills and preparations that are needed for a successful interview and match. Furthermore, Future research could attempt to directly state the important tips and steps that applicants can do to increase their chances and make them more prepared for the match cycle. Such direct information can include (1) how to prepare for an interview, (2) how to connect with mentors and research advisors, (3) how to prepare for electives and do well at them.

## Conclusions

This study provides comprehensive data about the factors that influence and attract program directors of orthopedic surgery in choosing their candidates for residency. Our hope is that medical students can use the data provided by this study to help them prepare and plan for the orthopedic residency match in Saudi Arabia. With the data provided by this study, applicants for orthopedic surgery have the advantage of early planning to build a strong application that may help persuade program directors to choose them.

## Data Availability

The data related to this study are available from the corresponding author upon reasonable request.

## Abbreviations

SCFHS: qSaudi Commission for Health Specialties
SMLE: Saudi medical license exam
GPA: Grade point average
LOR: Letters of recommendation
